# Supplementary material for: Three dimensional architected thermoelectric devices with high toughness and power conversion efficiency
Source: Nat Commun. 2023 Apr 12;14:2069. doi: 10.1038/s41467-023-37707-2 (PMC10097747; doi:10.1038/s41467-023-37707-2)
Supplement: Supplementary file 1 — Supplementary Information [file 41467_2023_37707_MOESM1_ESM.pdf]

Supporting Information

Three dimensional architected thermoelectric devices with high toughness and power conversion efficiency

Vaithinathan Karthikeyan[1,2ψ], James Utama Surjadi[3,4ψ], Xiaocui Li[3], Rong Fan[3], Vaskuri C. S. Theja[1,2], Wen Jung Li[3], Yang Lu[3,4,5]*, Vellaisamy A. L. Roy[6]*

[1]Department of Materials Science and Engineering, City University of Hong Kong, Kowloon, Hong Kong

[2]State Key Laboratory for Terahertz and Millimeter Waves, City University of Hong Kong, Kowloon, Hong Kong

[3]Department of Mechanical Engineering, City University of Hong Kong, Kowloon, Hong Kong

[4]Hong Kong Institute for Advanced Study, City University of Hong Kong, Hong Kong

[5]Department of Mechanical Engineering, The University of Hong Kong, Pokfulam, Hong Kong

[6]School of Science and Technology, Hong Kong Metropolitan University, Ho Man Tin, Hong Kong

* Corresponding Authors: Yang Lu (ylu1@hku.hk); Roy Vellaisamy (vroy@hkmu.edu.hk)

ψ These authors contributed equally to this work.

1. Deformation Mechanism

A simple buckling model consisting of a cylindrical shell filled with an elastic core is used to the calculate the dominant deformation mode in thermoelectric microlattices. The buckling stress (σ_{cr}) could be estimated as follows:

$$\boldsymbol{\sigma_{cr} = \frac{E_{TE}t}{r}f}$$

where E_{TE} and t represents the elastic modulus and thickness of the thermoelectric film used (i.e. Sb_2Te_3 or Bi_2Te_3), respectively. r represents the mean radius of the lattice strut, while f is defined by:

$$f = \frac{1}{12(1-v_{TE}^2)}\frac{(r/t)}{(\lambda_{cr}/t)^2} + \frac{(\lambda_{cr}/t)^2}{(r/t)} + \frac{3}{(3-v_c)(1+\ v_c)}\frac{E_c}{E_{TE}}(\frac{\lambda_{cr}}{t})(\frac{r}{t})$$

where E_c is the elastic modulus of the core (~ 3.6 GPa) obtained via uniaxial compression of partially carbonized PEGDA pillars under the same loading condition as the microlattice samples. v_{TE} and v_c represents the Poisson's ratio of the thermoelectric film and core, which was 0.3 and 0.4, respectively. The critical wavelength parameter, λ_{cr}, can be calculated by the following:

$$\frac{\lambda_{cr}}{t} = \left[\frac{(3-v_c)(1+\ v_c)}{12(1-v_{TE}{}^2)}\right]^{1/3}\left[\frac{E_{TE}}{E_c}\right]^{1/3}$$

where L is the length of the cylindrical strut, and m is the buckling mode. From nanoindentation, the obtained modulus of the thermoelectric film, E_{TE}, for Sb_2Te_3 and Bi_2Te_3 were ~ 105 GPa and ~ 68 GPa, respectively (Figure S8). Based on these, the calculated buckling wavelength parameter to thickness ratios, λ_{cr}/t, are plotted in Figure S9. Our composite microlattices lie well outside of the solid black line, indicating that the microlattices should possess a core that is sufficiently strong to support the thermoelectric films and result in a synergistic enhancement in load-bearing capability. This explains the ductile deformation behavior of the thermoelectric microlattices against the catastrophic or brittle failure observed in previous works (indicated by the drastic drop in stress level upon yielding) caused by the insufficient strength of the core. It has also experimentally demonstrated that at low r/t ratios, the core is not sufficiently stiff to support the shell and has little to no effect on the mechanical properties of the film. In this case, the shell effectively acts as a brittle hollow tube. It is only at a sufficiently high r/t ratio that the synergistic effect between a compliant core and stiff

shell could be leveraged, which results in enhanced buckling stress and deformability. Higher r/t ratio also reduces the stress concentration at the nodes, suppressing film fracture. Therefore, the thickness of the thermoelectric shell is crucial to maintain the deformability of the core-shell microlattices.

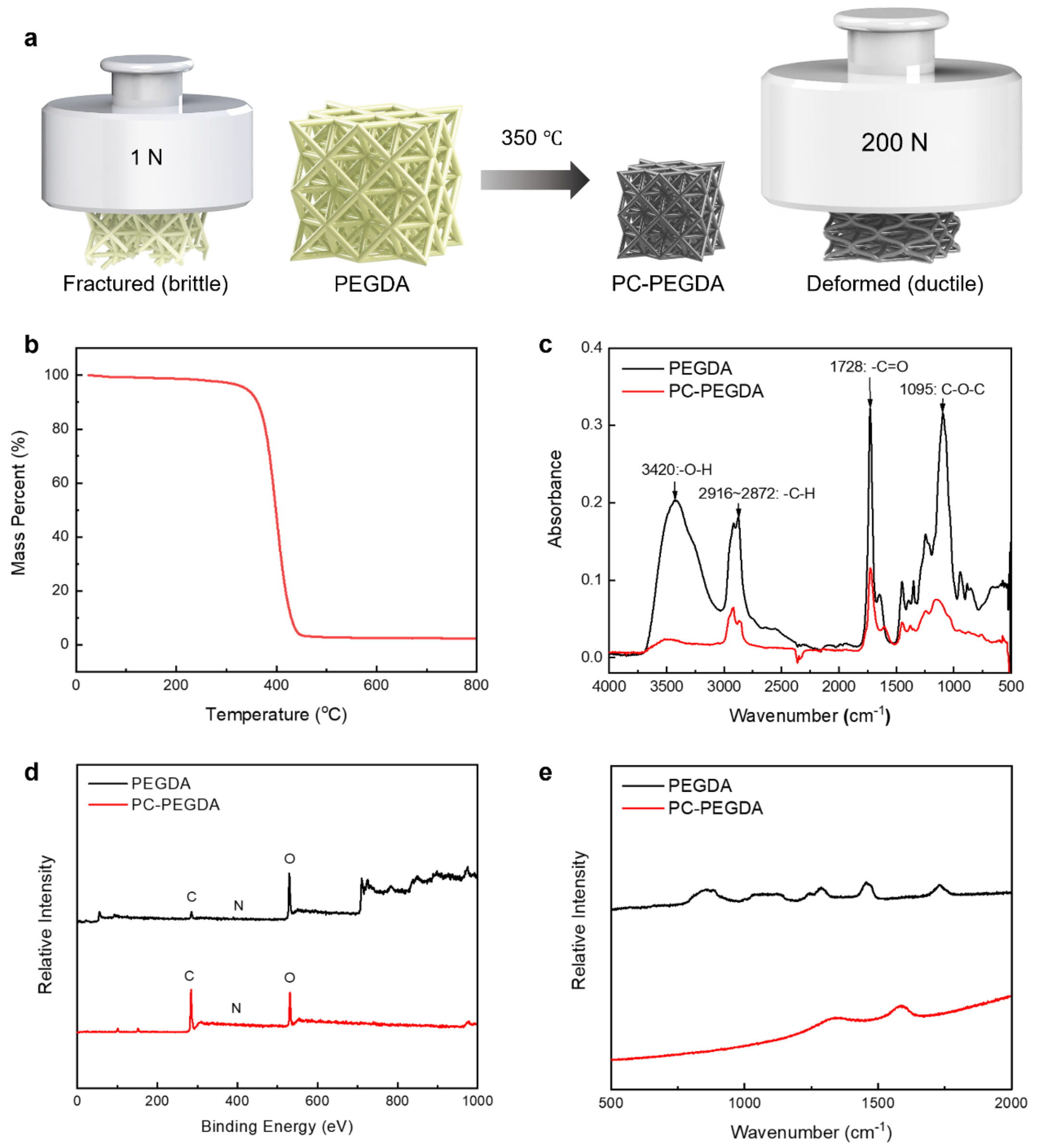

Figure S1 Materials characterization of the polymeric and partially carbonized microlattices. (a) Illustration of the key differences in mechanical properties between the polymer (PEGDA) and partially carbonized PEGDA (PC-PEGDA) microlattices. (b) Thermogravimetric analysis (TGA) of the polymer microlattices showing a degradation temperature in the range of 300 to 400°C. (c) Fourier Transform Infra-Red (FTIR) spectra showing the reduction of chemical bonds in the partially carbonized microlattices compared to as-fabricated polymer microlattices. (d) X-ray Photoelectron Spectroscopy (XPS) results exhibiting the significant increase in the carbon (C) to oxygen (O) ratio in the partially carbonized samples. (e) Raman spectra of the polymer and partially carbonized lattices, verifying the formation of amorphous carbon upon partial carbonization, as shown by the D-band and G-band peaks and weakening of characteristic polymer peaks.

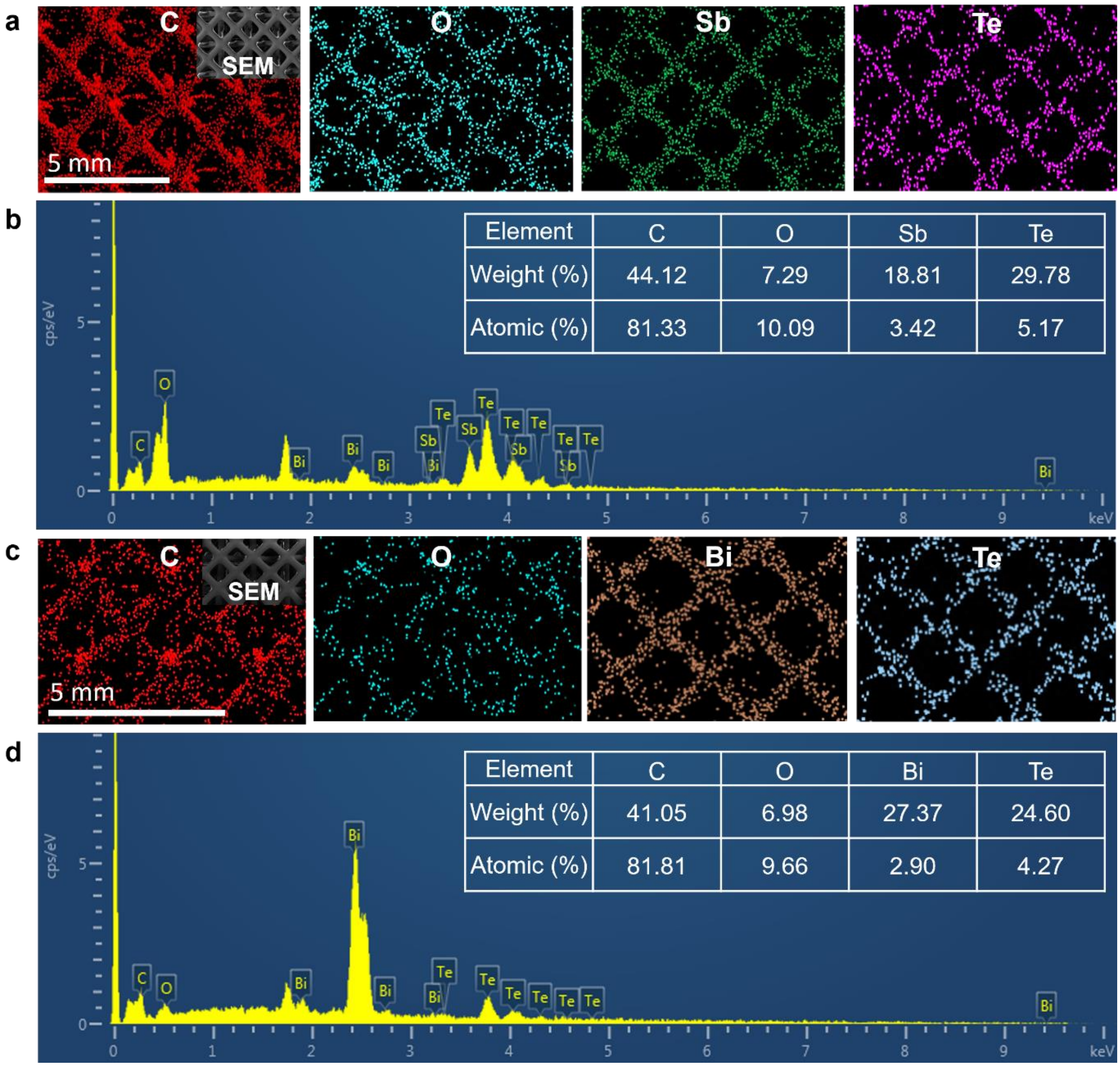

Figure S2 Energy Dispersive X-ray (EDX) spectroscopy of the thermoelectric films. (a) Elemental mapping of the antimony telluride-coated microlattice samples showing the homogeneous distribution of elements in the film. (b) Quantitative analysis of the ratio of elements from the antimony telluride-coated microlattices showing the desired ratio of Sb and Te elements (~2:3). (c) Elemental mapping of the bismuth telluride-coated microlattice samples showing the homogeneous distribution of elements in the film. (d) Quantitative analysis of the ratio of elements from the antimony telluride-coated microlattices verifying the desired ratio of Bi and Te elements (~2:3).

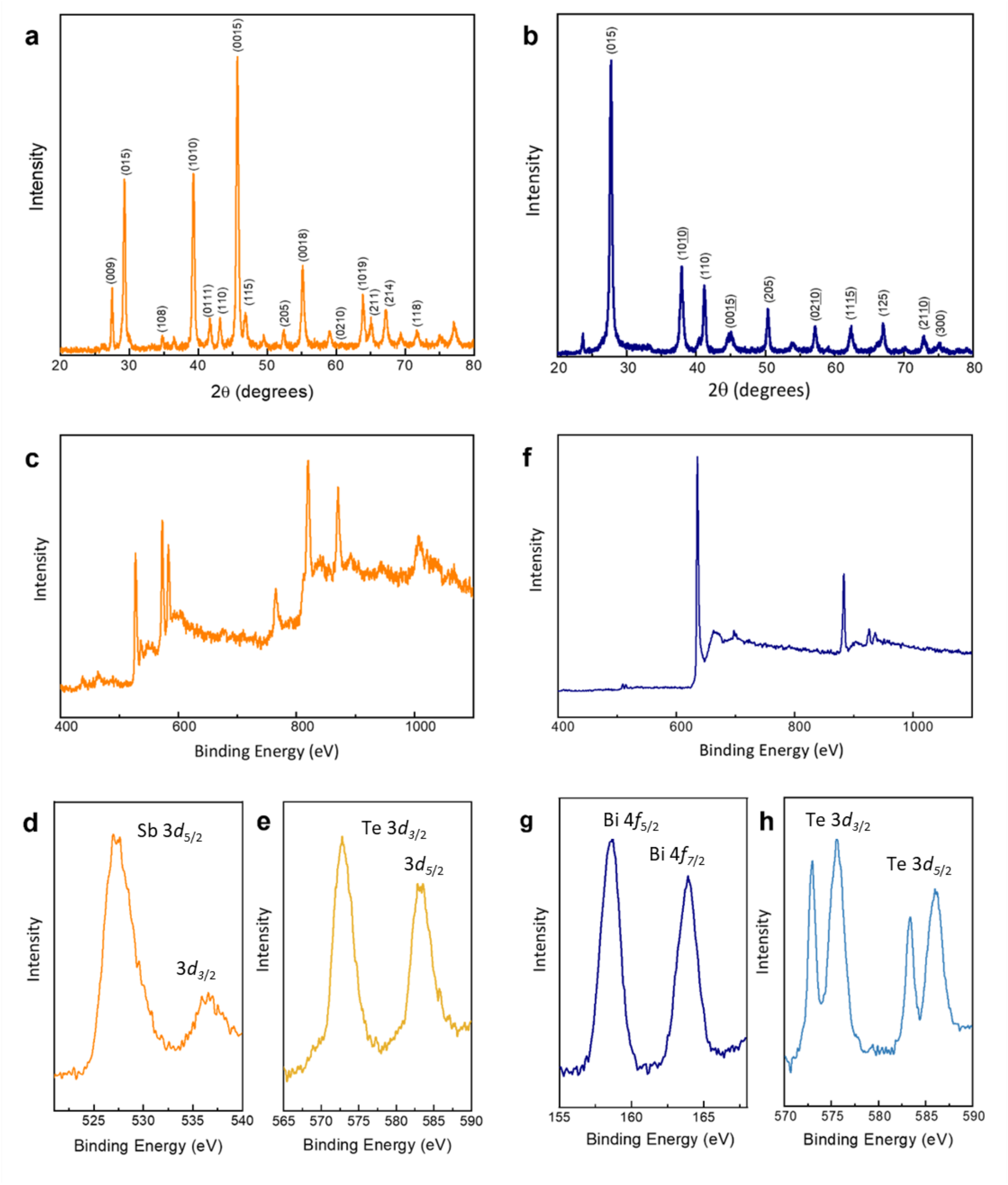

Figure S3 Detailed material characterization of the thermoelectric films. (a and b) X-Ray Diffraction (XRD) patterns obtained from the antimony telluride (a) and bismuth telluride films (b). (c to e) X-ray Photoelectron Spectroscopy (XPS) results of the antimony telluride films, confirming its crystalline structure with the desired chemical stoichiometry. (f to h) XPS spectra of the bismuth telluride films, which confirmed its crystalline structure with the desired chemical stoichiometry.

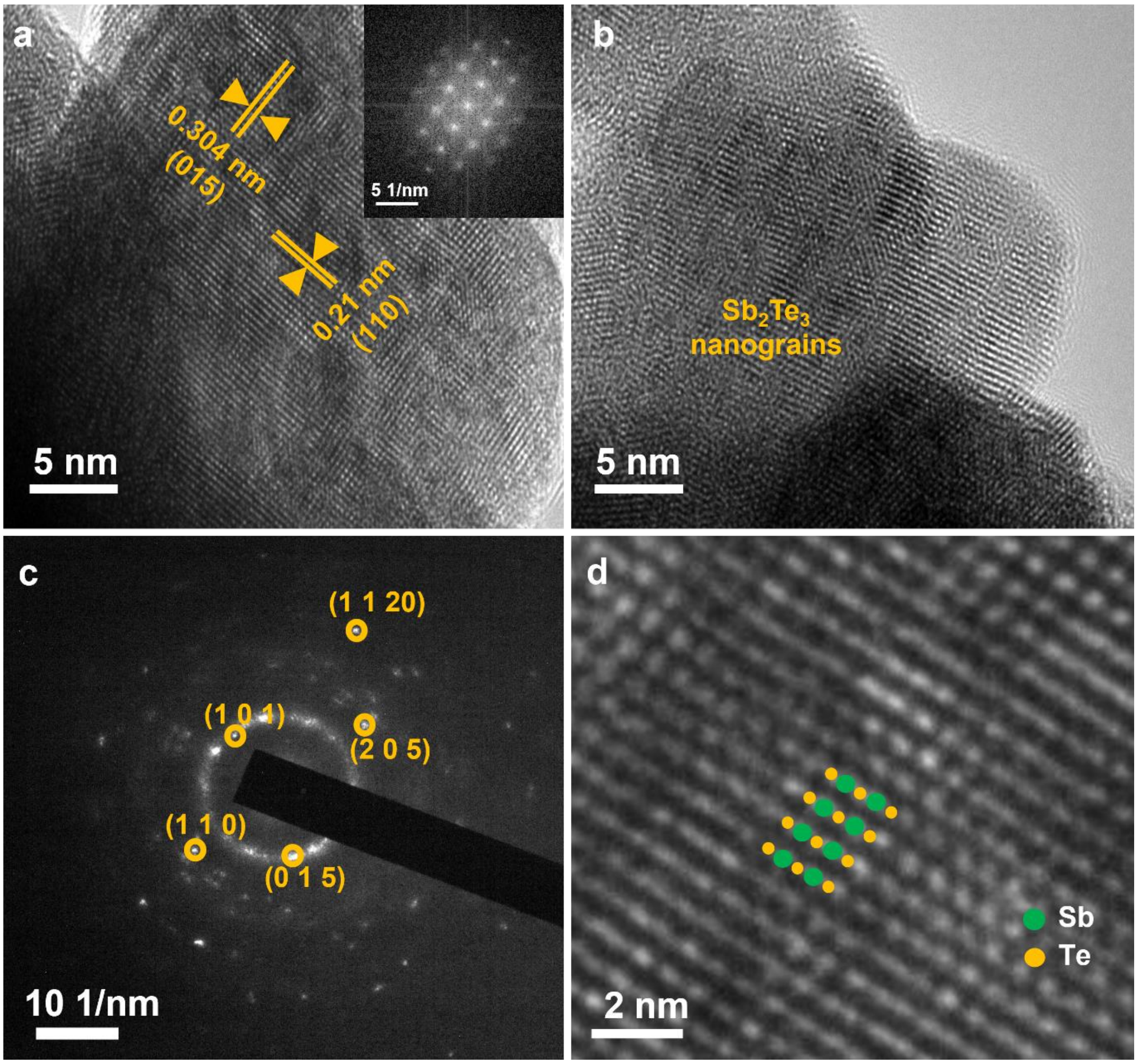

Figure S4 TEM analysis of the antimony telluride film. (a and b) High-resolution TEM (HRTEM) images of the antimony telluride film used for the p-type thermoelectric material, showing its nanocrystalline structure, lattice spacing, and orientation. (c) Selected area diffraction pattern (SAED) of the antimony telluride film showing its polycrystalline structure. (d) Magnified HRTEM image of the antimony telluride film showing the arrangement of Sb and Te atoms.

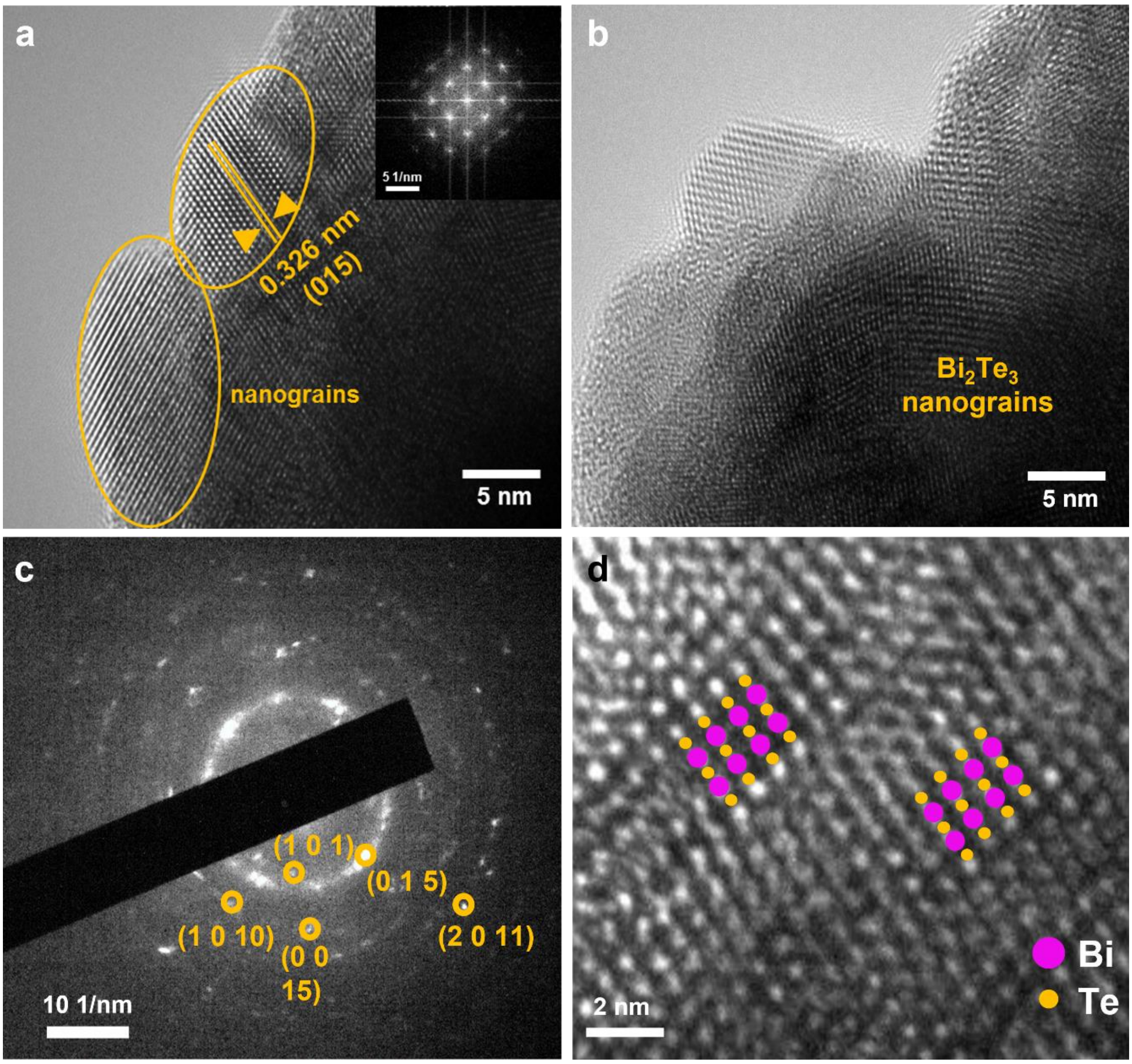

Figure S5 TEM analysis of the bismuth telluride film. (a and b) High-resolution TEM (HRTEM) images of the bismuth telluride film used for the n-type thermoelectric material, showing its nanocrystalline structure, lattice spacing, and orientation. (c) Selected area diffraction pattern (SAED) of the bismuth telluride film showing its polycrystalline structure. (d) Magnified HRTEM image of the bismuth telluride film showing the arrangement of Bi and Te atoms.

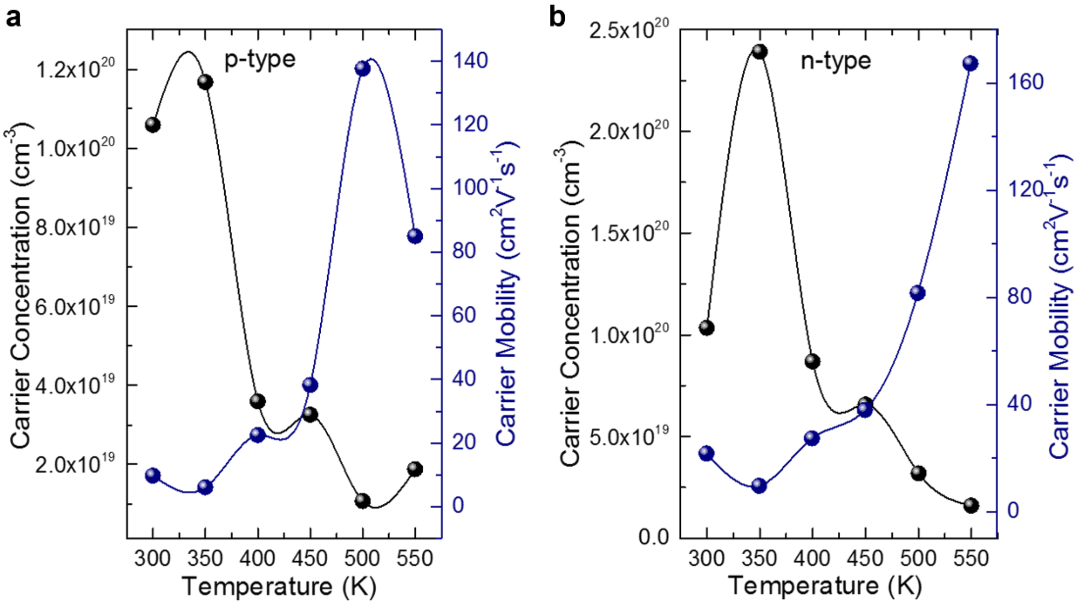

Figure S6 Thin-film transport properties of thermoelectric films. (a and b) Carrier concentration and carrier mobility for p-type (a) and n-type (b) materials at various temperatures.

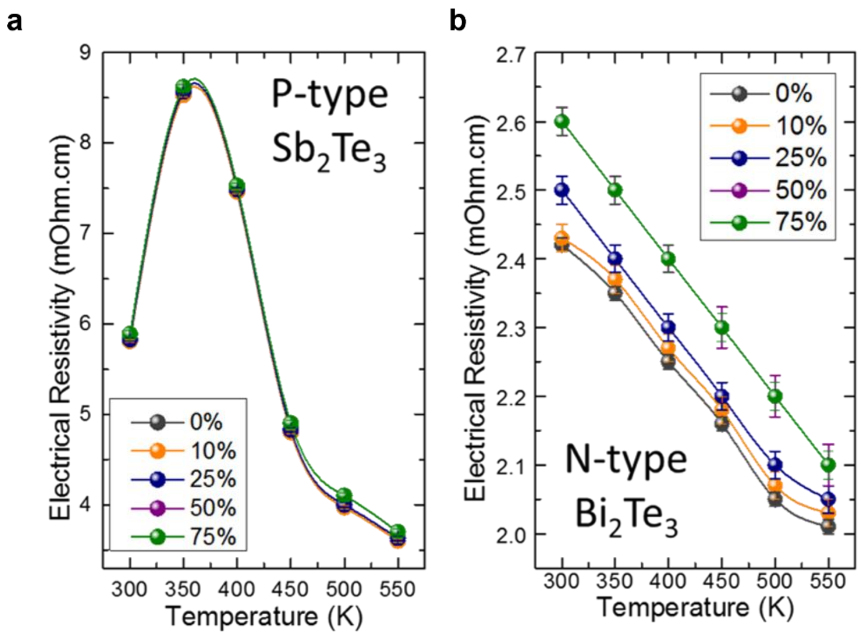

Figure S7 Mechanical strain-dependent electrical resistivity changes in 3D core-shell TE microlattice. Electrical resistivity versus temperature for the (a) p-type and (b) n-type microlattices after being deformed to various strain levels. Data are presented as mean while the error bar represents the standard deviation.

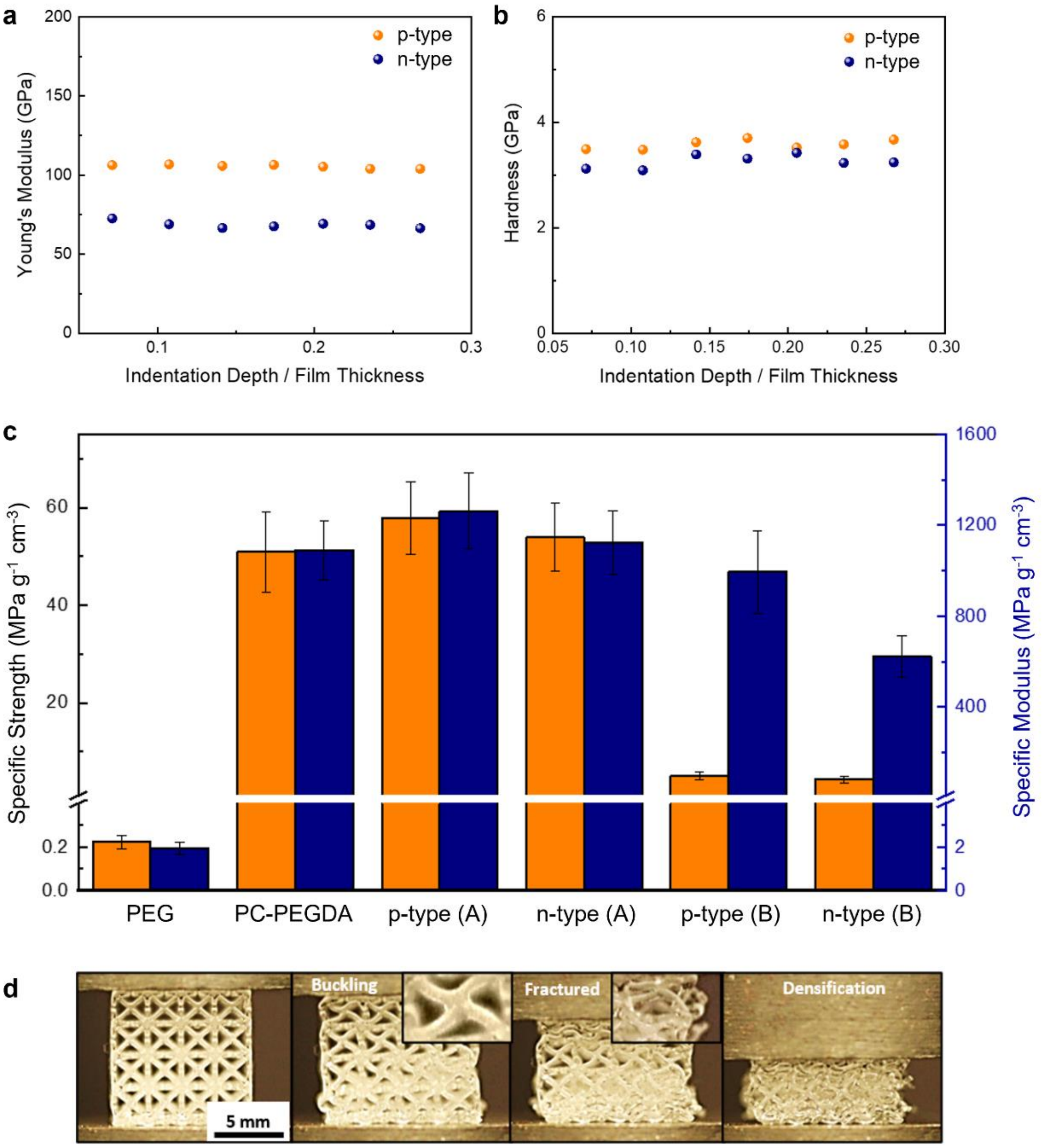

Figure S8 Mechanical analysis of thermoelectric films and microlattices. (a) Young's modulus and (b) Hardness of the thermoelectric thin films, antimony telluride (p-type) and bismuth telluride (n-type), tested at various indentation depths using nanoindentation testing. (c) Specific strength and specific modulus between the polymer, partially carbonized, and thermoelectric microlattices [p-type (A) and n-type (A)] compared to bulk thermoelectric legs [p-type (B) and n-type (B)]. (b) Deformation behavior of the polymer microlattice under uniaxial compression showing localized strut fracture upon the onset of buckling, followed by densification of fractured struts at higher compressive strains. Data are presented as mean while the error bar represents the standard deviation.

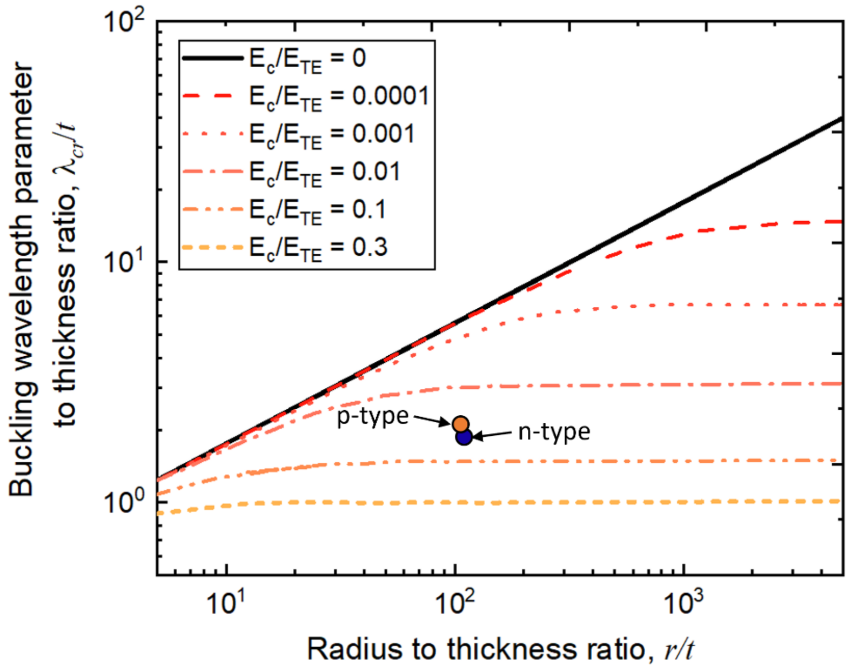

Figure S9 Buckling wavelength parameter to thickness ratios plotted against radius to thickness ratio.

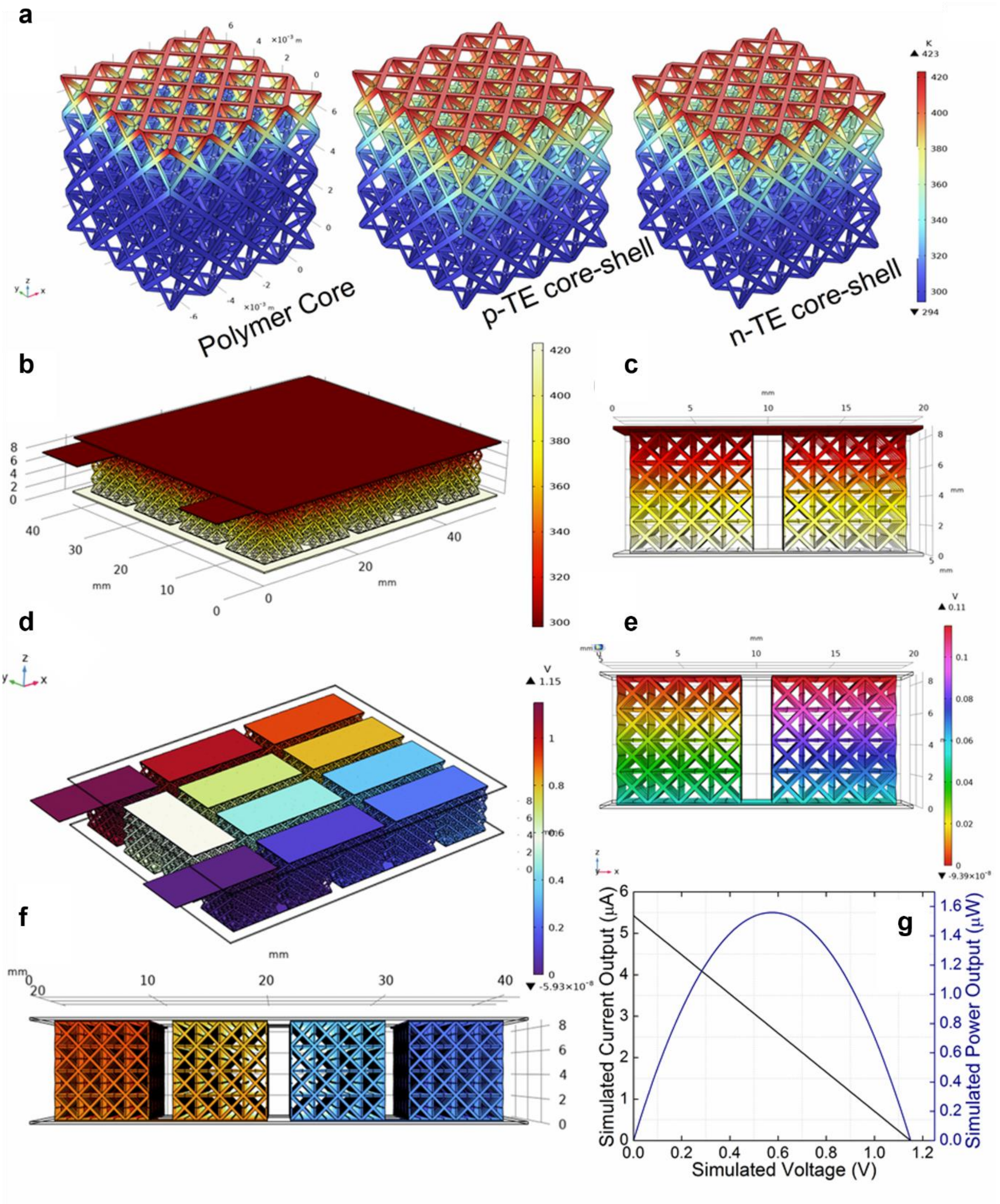

Figure S10 COMSOL Multiphysics simulation for 3D core-shell TE microlattice structures and devices. (a) Thermal conduction distribution of polymer core without TE shell and with p- and n-TE shell structure (b) and (c) simulated thermal gradient distribution of 3D thermoelectric whole device and one TE leg pair (d),(e) and (f) simulated voltage generation profile of 3D thermoelectric whole device and one TE leg pair (g) simulated power characteristics of 3D TE core-shell microlattice structure at a thermal gradient of 120℃.

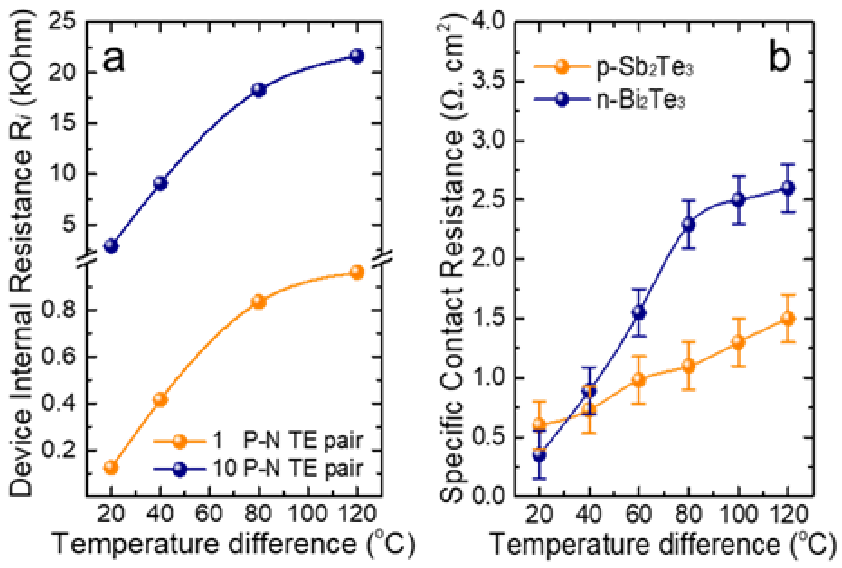

Figure S11 Change in Device Resistance with temperature (a) Total internal resistance of 3D p-n pair (b) Specific contact resistance of 3D p- and n- TE microlattice structures. Data are presented as mean while the error bar represents the standard deviation.

Table S1 Comparison of our 3D core-shell TE microlattice work with state-of-the-art 3D printed TE devices and commercial TE devices.

**Method**	**TE Materials**	**TE Leg Size**	**ΔT (K)**	**V_{oc} (mV)**	**ω_{max} (W/cm^2)**	**Remarks**
3D Direct Ink Writing Kim et al[7]	p-$Bi_{0.5}Sb_{1.5}Te_3$ n-$Bi_2Te_{2.7}Se_{0.3}$	350 µm	83	40	479µ	Porous structure with poor mechanical strength and hard to sustain larger ΔT.
3D Extrusion printing Kim et al[9]	p-$Bi_{0.4}Sb_{1.6}Te_3$ n-$Bi_2Te_{2.7}Se_{0.3}$	10 mm	40	27	1.42 m	3D printed bulk TE leg structures and hard to sustain larger ΔT.
3D Shape Conformable Thermoelectric paint[63]	Sb_2Te_3(P) Bi_2Te_3(N)	~25 mm	30	30	4m	Painting on 3D surface with thermoelectric ink for increasing thermal efficiency.
Commercial TEG[65]	Sb_2Te_3(P) Bi_2Te_3(N)	~3 mm	230	0.5	2.1	Commercial Bulk TEG device with ~120 P-N leg pairs.
Dispenser Printing Chen et al[64]	Sb_2Te_3(P) Bi_2Te_3(N)	640 µm	20	320	75µ	2D planar device with 50 p-n leg pairs.
2D Thin film µTEG Vieira et al[66]	Sb_2Te_3(P) Bi_2Te_3(N)	400 nm	35	210	3.3m	Co-evaporation fabricated 15 p-n TE leg pairs
Screen Printed flexible TEG Kim et al[67]	Sb_2Te_3(P) Bi_2Te_3(N)	~500 µm	50	85	3.5m	Demonstrated wearable flexible TEG using glass fabric as scaffold
Wearable TEG Hong et al [68]	Sb_2Te_3(P) Bi_2Te_3(N)	5 mm	10	1.75	25 µ	Wearable bulk TEG with thermoelectric pillars
Wearable TEG C.S Kim et al[69]	Sb_2Te_3(P) Bi_2Te_3(N)	2.5 mm	10	85	38µ	Wearable bulk TEG with 160 pairs of commercial TEG legs
Screen printed flexible TEG Kim et al [70]	p-$Bi_{0.5}Sb_{1.5}Te_3$ n-$Bi_2Te_{2.7}Se_{0.3}$	~600 µm	26	700	6.32m	Ionized defect engineering processed screen-printed BiTeSe thick film device
3D Core-Shell TE microlattice (This work)	Sb_2Te_3(P) Bi_2Te_3(N)	8 mm	120	1100	1.45m	3D Core-shell TE devices were realized with larger efficiency due to controlled heat stagnation and larger thermal gradient in the TE legs.
